# Focus on Extremely Late Relapses in Hodgkin Lymphoma: A Single Center Analysis

**DOI:** 10.3390/cancers18050777

**Published:** 2026-02-28

**Authors:** Athanasios Liaskas, Fotios Panitsas, Maria K. Angelopoulou, Marina Siakantaris, Gerassimos A. Pangalis, Theodoros P. Vassilakopoulos

**Affiliations:** 1 Department of Hematology and Bone Morrow Transplantation, “Laikon” General Hospital, National and Kapodistrian University of Athens, 11527 Athens, Greece; ath.liaskas@gmail.com (A.L.); fpanits@gmail.com (F.P.); mkangelop@gmail.com (M.K.A.); siakantaris@gmail.com (M.S.); 2 Athens Medical Center, “Psychikon” Branch, 11525 Athens, Greece; pangalis@med.uoa.gr

**Keywords:** extremely very late relapses, Hodgkin lymphoma

## Abstract

**Background/Objective**: Very late relapses (VLRs) in Hodgkin lymphoma (HL), occurring ≥5 years after initial treatment, have been well-recognized for decades. Recent studies have highlighted the persistent and linear risk of relapse extending well beyond 5 years, while also describing the incidence and risk factors. However, extremely late relapses (EXLRs) occurring ≥20 years after diagnosis remain poorly described and largely limited to case reports. In the present study, we focus on patients experiencing EXLRs after chemotherapy or combined modality therapy, aiming to define their incidence, clinical characteristics, and outcomes, and to compare them with patients with VLRs relapsing between 5 and 20 years from initial diagnosis. **Methods:** We performed a retrospective study of 270 patients with HL, who received chemotherapy ± radiotherapy (CT ± RT), remained in continuous remission at least 5 years from treatment initiation and had follow-up data exceeding 20 years from diagnosis. The primary endpoint was the cumulative incidence (CumInc) of VLR, considering death from any cause as a competing event. **Results:** EXLR occurred in 9/270 cases. The CumInc of EXLR at 25, 30 and 35.2 years was 2.23%, 4.80% and 7.87%, respectively. Compared to patients with VLRs (N = 61), patients with EXLRs had a lower incidence of anemia and a higher incidence of lymphocytopenia at diagnosis. At relapse, patients with EXLR tended to be older, had a lower frequency of extranodal disease, were more often managed with the same or similar salvage CT regimen as at initial diagnosis (reinduction), and were considered less frequently as candidates for autologous stem cell transplantation. In univariate analysis, the omission of RT at initial treatment and the histologic subtype of mixed cellularity (MC) were associated with a higher probability of EXLR. Compared to patients with VLRs, patients with EXLRs had a 10-year treatment to second failure (TT2F) of 88.9% versus 47.4%, a 10-year overall survival after relapse (O2S) of 77.8% versus 54.9% and a 10-year disease-specific survival (DSS) of 88.9% versus 70.9%. **Conclusions:** The study describes for the first time the incidence and characteristics of EXLRs in HL. These patients had excellent outcome despite having relapsing disease, suggesting that this subgroup of patients may have distinct biological characteristics.

## Simple Summary

Our study examined extremely late relapses (EXLRs) in Hodgkin lymphoma (HL), defined as relapses occurring after at least 20 years from initial diagnosis. We reviewed 270 patients with HL in continuous remission for at least 5 years and with follow-up data exceeding 20 years from initial diagnosis. Nine patients experienced EXLR. The cumulative incidence of EXLR gradually increased over time. Patients with EXLR generally had more favorable characteristics at relapse and were treated less aggressively compared to patients who relapsed between 5 and 20 years. Omission of radiotherapy at initial treatment and the histologic subtype of mixed cellularity were associated with a higher probability of EXLR. Patients with EXLR achieved a favorable disease control and survival after relapse, suggesting that these late relapses may represent a biologically different and less aggressive form of disease.

## 1. Introduction

In Hodgkin lymphoma (HL), very late relapses (VLRs) are defined as disease recurrences occurring more than 5 years after the initiation or completion of the first-line treatment. VLRs have been recognized since 1984, primarily through case reports, small case series, and moderately sized cohorts [1,2,3,4,5,6,7,8,9,10,11,12,13]. Extremely late relapses (EXLRs), occurring beyond 20 years and up to 30–35 years after diagnosis, have also been documented in case reports including patients relapsing after 24, 29 and 30 years as well as two patients relapsing after 32 years, all treated with monotherapy with RT [3,7,14,15]. Since 2017, three major studies have been published aiming to systematically address the issue of VLRs. The German Hodgkin Study Group (GHSG) first reported on the incidence of VLRs within the HD7-HD12 randomized trials, among patients treated with combined modality approaches. Their study demonstrated that patients with HL who remained in first complete remission (CR1) for more than 5 years after treatment initiation with chemotherapy with or without radiotherapy (CT ± RT) remain at risk of recurrence at least for the subsequent 15–20 years, and the cumulative incidence (CumInc) of VLR rises in a linear pattern over time. Also, male gender and early stages were independent predictors of higher CI. However, the follow-up time did not exceed 20 years after the initial diagnosis [16]. In 2022, Andersen et al. presented the results of their population-based study including 2.424 patients with HL derived from the Danish National Lymphoma Registry, and with a maximum follow-up time of 25.5 years. They reported nodular lymphocyte predominance (NLPHL) and mixed cellularity (MC) histological subtypes (in females) as risk factors for VLR, along with age and lymphocytopenia [17]. The latest VLR was recorded at 18.9 years after the initial diagnosis. Simultaneously, we presented the results of our study, reporting a slightly higher incidence than GHSG and confirming the linear pattern of relapse, further extending this observation and demonstrating that the linear trend of VLRs continues beyond 20 years from diagnosis up to 25 or even 30 years. We also described risk factors for developing VLR, including age, MC histology, low erythrocyte sedimentation rate (ESR), omission of RT and the non-anthracycline-containing regimens in first-line treatment [18]. All these data are summarized in reference [19].

In the present study, we focused on patients with EXLR occurring 20 years or more after initial treatment initiation with chemotherapy or combined modality therapy. We aimed to describe the incidence of EXLR in patients who had already reached 20 years in the first CR, describe such patients’ clinical characteristics and outcomes and compare them with those experiencing VLR but not EXLR (i.e., those relapsing beyond 5 but earlier than 20 years from the initial diagnosis).

## 2. Materials and Methods

### 2.1. Patients

Among 1842 consecutive patients with HL treated between 1976 and 2020, 23 received RT alone and 1819 received CT ± RT. Among these 1819 patients, 1253 were alive and disease-free at 5 years following the initiation of CT, among whom 70 developed VLRs and 95 died of benign or malignant causes unrelated to HL while in 1st CR (CR1). Overall, a subgroup of 270 patients had follow-up data exceeding 20 years from diagnosis and were eligible for analysis, and 9/270 developed EXLRs, while 22/270 died of benign or malignant causes unrelated to HL while in CR1.

### 2.2. Treatment Strategies

Between 1980 and 1987, early-stage patients were offered combined modality therapy (CMT) including low-dose involved-field RT and MOPP chemotherapy, with a minority receiving MOPP-equivalents, such as ChlVPP (chlorambucil, vinblastine, procarbazine, prednisone) or COPP (cyclophosphamide instead of nitrogen mustard). Between 1988 and 1998, early-stage patients received EBVD (with epirubicin instead of adriamycin, bleomycin, vinblastine and dacarbazine), considered as ABVD-equivalent. Roughly after 1998, all patients were treated with ABVD. RT was routinely administered to early-stage patients, being omitted in a minority based on the patient’s preference or medical contraindications. All advanced-stage patients received MOPP or equivalents until 1980. Thereafter, from 1981 until 1997, advanced-stage patients were successively treated with alternating MOPP/ABVDx12, MOPP/ABV or MOPP/EBV hybridx6, alternating MOPP/ABVDx6 or even EBVD. Beyond 1998, ABVD became the standard regimen. RT was not administered to advanced-stage patients except for a small minority, mainly for suspected residual disease. None of the patients received any form of consolidative therapy other than chemotherapy or combined chemotherapy and radiotherapy, which constituted the standard first-line therapy. No interim PET-based strategy was used in this patient population, as this was adopted in 2008 and the follow-up time was <20 years [18].

### 2.3. Statistical Analysis

Data on demographic characteristics, initial and salvage therapy and patients’ characteristics at the time of diagnosis and VLR were extracted from patients’ medical records. Variables evaluated at diagnosis and relapse included: age, gender, presence of B-symptoms, extranodal involvement, anemia (hemoglobin <13.0 g/dL for males and <11.5 g/dL for females) [20] and Ann Arbor stage, as well as treatment strategy and intention to proceed to ASCT. Median values, range and interquartile range (IQR) were used to summarize continuous variables and frequencies and percentages for categorical variables. Comparisons between categorical variables were performed with Pearson’s chi-square test. The primary endpoint was the CumInc of EXLR after the landmark time of 240 months from treatment initiation, considering death from any cause without prior relapse as a competing risk. Patients who were alive in continuous first remission were censored at last follow-up. Aalen–Johansen curves for the competing risks of relapse and death in first remission were estimated [21].

Time to second treatment failure (TT2F) was defined as the time interval between salvage therapy initiation and treatment failure (defined as toxic death during salvage therapy or failure to achieve complete or partial remission) or second relapse or last date of follow-up in second complete remission (CR2). Deaths of any cause in CR2 were censored for the analysis of TT2F. Overall survival after relapse (O2S) was defined as the time interval between salvage therapy initiation and death of any cause or last date of follow-up. Disease-specific survival after relapse (DSS) was defined as the time interval between salvage therapy initiation and death, excluding deaths from unrelated benign causes.

Statistical analysis was performed with mstate package (Cuminc function) and Epi package in R (version 4.5).

## 3. Results

### 3.1. Incidence of Extremely Late Relapses

**Figure 1:**
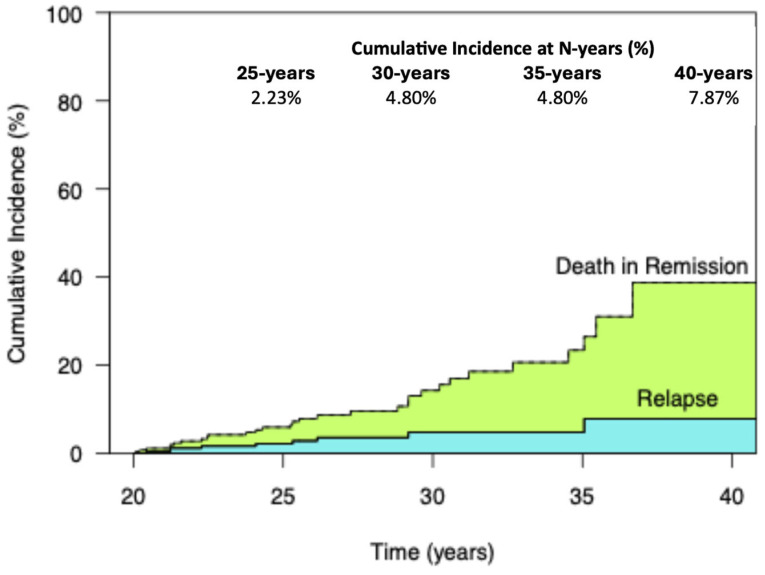
Cumulative incidence of EXLR and competing risk of death in patients who remain in first complete remission for 20 years after initiation of combined modality treatment.

Among 270 patients who remained in CR1 for >20 years from first-line treatment initiation, 9 patients eventually relapsed. The CumInc (±standard error) of EXLR at 25, 30 and 35.05 years for the patient population was 2.23 ± 0.99%, 4.80 ± 1.80% and 7.87 ± 3.48%, respectively. At 35 years, it remained at 4.80 ± 1.80% (Figure 1).

### 3.2. Patients’ Characteristics

Patients’ characteristics at diagnosis and relapse are presented in Table 1. Compared to the 61 patients with VLRs who had relapsed >5 but <20 years from initial treatment initiation, the 9 patients with EXLRs had very similar characteristics at the initial diagnosis, with the exception of a lower incidence of anemia and a higher incidence of marked lymphocytopenia (*p* = 0.11 for both, Table 2). Regarding characteristics at relapse, patients with EXLRs expectedly had older age at relapse (≥65 years old 44.4% vs 18.0%, *p* = 0.071), but also had more favorable characteristics, especially an absence of extranodal disease (0% vs. 27.6%, *p* = 0.088) and also more frequent reinduction (37.5% vs 13.1%, *p* = 0.076) and no intention of undergoing second-line autologous stem cell transplantation (0% vs. 23.0%, *p* = 0.108), as shown in Table 2.

Regarding histology, five patients had MC, three had NS and one had NLP at diagnosis. Among the five patients with MC, one had MC and one had unclassified classical HL at the time of EXLR, one had no data available and two had NS. Among the two latter patients, histology review was possible both at diagnosis and EXLR, and indeed, there was discordance with the transition from MC to NS, pointing to the possibility of the development of a new, unrelated HL. Among the three cases with NS at diagnosis, one had NS and one had unclassified classical HL at the time of EXLR, while the last patient had NLP at EXLR. Given that the initial NS diagnosis was made in 1988, at a time when NLP had not yet been recognized, it is very likely that the original diagnosis could have been NLP from the beginning. The last patient had NLP both at baseline (on retrospective review) and at the time of EXLR, although she had been diagnosed with NS at another center in 1996. Overall, there was a strong suspicion of the development of a new HL in one case and possibly in the other MC-to-NS transition, although a review of the original specimen was not possible.

### 3.3. Prognostic Factors for the Development of Extremely Late Relapses

In univariate analysis, age ≥ 45, gender, stage, B-symptoms, anemia, elevated ESR ≥ 50 mm/h, leukocytosis, marked lymphocytopenia and low serum albumin did not hold any prognostic significance, albeit the hazard ratios on competing risk analysis were 0.28 (95% CI 0.03–2.23, *p* = 0.23) for the presence of anemia (protective), 0.38 (95% CI 0.08–1.91, *p* = 0.24) for the presence of elevated ESR ≥ 50 mm/h (protective) and only 1.62 (95% CI 0.34–7.71, *p* = 0.54) for age ≥ 45. However, the omission of RT at initial treatment and the histologic subtype of MC were associated with a higher probability of EXLR, with hazard ratios on competing risk analysis of 3.46 (95% CI 0.95–12.70, *p* = 0.061) and 0.317 (for receipt of RT, 95% CI 0.09–1.15, *p* = 0.08), respectively. Multivariate analysis was not attempted due to the small number of events.

According to our previously published risk score (age ≥ 45 years old, no RT, MC histology and ESR < 50 mm/h), 215/270 patients had full data at diagnosis and 8/215 developed EXLRs (3.7%). The frequency of EXLR was 0/51 for patients with no risk factors, 4/102 (3.9%) and 1/44 (2.3%) for those with 1 or 2 risk factors, respectively, and 3/18 (16.7%) for patients with 3–4 factors (2/16 and 1/2 for 3 and 4 factors) (*p* < 0.001).

### 3.4. Outcome of Extremely Late Relapses

Patient #6 was 81 years old at the time of relapse and died of disease and complications within 1.21 months. All the eight remaining patients received conventional chemotherapy with curative intent with or without RT and achieved a complete second remission (CR2), as shown in Table 1. Patient #4 relapsed at the age of 71 and died in CR2 of secondary gastric cancer outside of any RT field at 28.9 months, while the remaining seven patients remained in CR2 for a median of 84.6 months (range 29.3–132.1).

Compared with the 61 patients with VLR but not EXLR, the nine patients with EXLRs had a 10-year TT2F of 88.9% versus 47.4% (*p* = 0.07), a 10-year O2S of 77.8% versus 54.9% (*p* = 0.57) and a 10-year DSS of 88.9% versus 70.9% (*p* = 0.49).

## 4. Discussion

In the present study, we identified 9 patients with EXLRs among 270 patients with HL, who had remained in CR1 for 20 years after the initiation of first-line therapy. These patients represented a minority of the 70 patients with VLRs. The focus of this analysis on EXLRs is unique in the literature, as such cases have been reported only as isolated cases or small case series. Interestingly, in two of the three large reports of VLRs, follow-up was capped at 20 years in the German study, while the last relapse was observed at 16.9 years in the Danish one [16,17].

Consistently with our previous reports on VLRs [18], there appears to be a more or less linear pattern of relapse, even after 20 years and up to at least 30 years, with the latest relapse observed at 35.2 years from treatment initiation. This was seen using competing risk analysis, which is crucial in studies with a long follow-up, where unrelated deaths may prevent the appearance of a future VLR. This competing risk analysis demonstrated a 2.2%, 4.8% and 7.9% mortality at 25, 30 and roughly 35 years in patients who were in CR at 20 years, with 9 EXLRs and 22 unrelated deaths. Compared to Kaplan–Meier analysis when censoring the unrelated deaths, the estimate was slightly lower (2.3%, 5.0% and 8.8). Due to the low number of events, we were not able to identify statistically significant prognostic factors for the appearance of EXLR. Notably, MC histology and the absence of RT in the initial treatment, which had been identified as risk factors for VLRs [18], were of marginal significance, with hazard ratios in the order of 3 in the prediction of EXLRs. Baseline anemia and ESR ≥ 50 mm/h had a statistically weaker protective effect, with hazard ratios of 0.28 and 0.38 (corresponding to 3.6 and 2.6), while age ≥ 45 years had almost no impact on the prediction of EXLR. Given the paucity of events, multivariate analysis was not performed. However, our previously published risk score (age ≥ 45 years old, no RT, MC histology and ESR < 50 mm/h) did affect the frequency of EXLRs despite the inability to show the significance of individual factors, even in univariate analysis.

Comparing the baseline characteristics of the 9 patients with EXLRs and the 61 patients with VLRs but not EXLRs, no differences were observed. However, there were potentially important numerical differences regarding the characteristics at the time of relapse, as shown in Table 2. In particular, patients with EXLRs tended to be older at relapse and were less likely to have extranodal and advanced-stage disease and anemia. Also, patients with EXLRs were more often treated with the same or similar salvage CT regimen as at initial diagnosis (reinduction) and were considered less frequently as candidates for autologous stem cell transplantation.

Regarding histology, only 3/8 cases both with available baseline and EXLR data had full concordance, with 1 case of MC, NS and NLP each. In one case, there was a confirmed transition from MC to NS. Another similar case could not be verified in the absence of available baseline tissue. The two cases of MC and NS that were diagnosed as unclassifiable at relapse are not informative regarding the identity of the clonal process, while the NS case that developed NLPHL is most likely a misdiagnosis at baseline in 1988. In the large GHSG series of VLRs [16], which, however, did not include an EXLR, the concordance between diagnosis and VLR was 68% and 70% for NS and MC at baseline. However, 10% of MCs had a transition to NS and 5% of NSs to MC. Furthermore 5% and 7% of NS and MC were diagnosed as lymphocyte-rich classical HL at VLR, while 20% and 13% of NS and MC cases relapsed as classical HL unclassifiable. Thus, the histologic discrepancies reported here for EXLRs were not unexpected but appear to be more frequent compared to those observed in VLR, occurring up to 20 years from diagnosis. Obviously, the year of diagnosis, which was earlier than the publication of the REAL classification in 1994 in 6/9 cases and prior to the WHO in 8/9, may also have a role on the interpretation of these observations.

Interestingly, excluding one very elderly patient who died almost immediately, all the remaining eight patients achieved a second CR and still remained disease-free at their last follow-up for a median of at least 7 years, with one of them having succumbed to gastric cancer unrelated to previous RT at the age of 71. Although the difference was not statistically significant, this very high rate of freedom from second treatment failure is in sharp contrast with the almost 50% rate observed in the overall VLR population [18]. This might be related to the origin of EXLRs. Thus, we could speculate that EXLRs were more likely to be clonally unrelated “new HL” than VLRs occurring earlier, and thus more likely to carry the favorable prognosis of a previously untreated HL. Assessment of the clonal relationship between diagnosis and relapse has been performed in patients with HL, using microdissected Hodgkin–Reed cells from formalin-fixed, paraffin-embedded tissue specimens. However, this analysis has not been performed specifically in patients with VLRs or EXLRs and our hypothesis remains to be tested [22,23].

## 5. Conclusions

In conclusion, the present study describes for the first time the incidence and characteristics of EXLRs in HL. These patients had excellent outcomes despite having relapsing disease, suggesting that this subgroup of patients may have distinct biological characteristics. EXLR after pure ABVD, BEACOPP-esc or novel agent-based strategies should be further evaluated as such strategies mature, to provide additional information [24]. Larger international cohorts are required to validate our findings and draw robust conclusions about the underlying pathophysiology and optimal management.

## Figures and Tables

**Table 1:** Clinical characteristics of patients with extremely late relapses.

Case	Age at Diagnosis/Relapse (Years)	Gender	Histology and Year of Diagnosis	Stage at Diagnosis	TTR (Years)	Histology at Relapse	Stage at Relapse	Initial Treatment	Salvage Treatment	CR2 (mo)
1	36/71	F	MC/1982	IIBX	35.05	MC	IIIB	MOPP-Bleox11	ABVDx8	99.4+
2	30/56	F	NS/1988	IA	26.14	NLP	IIIA	MOPPx5 + RT	R-CHOPx6	64.4+
3	28/57	M	MC/1990	IA	29.18	NS	IA	EBVDx5 + RT	ABVDx2 + RT	64.7+
4	47/71	M	MC/1992	IVB	24.09	Uncl	IA	MOPP/ABV	ABVDx4 + RT	28.9 *
5	20/45	F	NS/1989	IIIBX	25.32	NS	IIA	MOPP/ABVD	ABVDx6 + RT	132.1+
6	59/81	M	MC/1991	IVB	22.27	NA	NA	MOPP/ABV	No treatment	-
7	44/65	F	NLP/1996	IA	21.22	NLP	IA	ABVDx6 + RT	R-CVPx8	84.6+
8	35/55	M	MC/1997	IA	20.43	NS	IB	ABVDx5 + RT	ChlVPP/ABVx4 + RT	82.7+
9	34/55	M	NS/2001	IIIA	21.22	Uncl	IIA	ABVDx6 + RT	BV-ESHAPx3 + BV	29.3+

M: male; F: female; TTR: time to relapse; CR2: second complete remission; MC: mixed cellularity; NS: nodular sclerosis; NLP: nodular lymphocyte predominant; Uncl: unclassified; NA: not available; MOPP: mechlorethamine, vincristine, procarbazine, prednisone; EBVD: epirubicin, bleomycin, vinblastine, dacarbazine; ABVD: adriamycin, bleomycin, vinblastine, dacarbazine; Bleo: bleomycin; RT: radiotherapy; R-CHOP: rituximab, cyclophosphamide, doxorubicin, vincristine, prednisone; R-CVP: rituximab, cyclophosphamide, vincristine, prednisone; ChlVPP: chlorambucil, vinblastine, procarbazine, prednisone; BV-ESHAP: brentuximab vedotin, etoposide, cytarabine, cisplatin, methylprednisolone. * Death of gastric cancer outside any RT field.

**Table 2:** Comparison of characteristics of patients with very late and extremely late relapse.

Patients’ Characteristics at Diagnosis	VLRs but Not EXLRs (5–20 Years)		EXLRs (>20 Years)		*p*-Value
Age ≥ 45 years	21/61	34.4%	2/9	22.2%	0.47
Male gender	39/61	63.9%	5/9	55.6%	0.63
Iliac/inguinal involvement	16/61	26.2%	2/9	22.2%	0.30
Stage I/IIA vs. I-IIB/III/IV	31/61	50.8%	5/9	55.6%	0.79
B-symptoms	24/61	39.3%	4/9	44.4%	0.77
IPS ≥ 3	10/54	18.5%	2/7	28.6%	0.53
MC histology	26/59	44.1%	5/9	55.6%	0.52
Anemia	23/60	38.3%	1/9	11.1%	0.11
Lymphocytopenia	1/55	1.80%	1/8	12.5%	0.11
Albumin < 4 g/dL	20/43	46.5%	4/6	66.6%	0.36
ESR ≥ 50 mm/h	15/50	30.0%	2/8	25.0%	0.77
RT in the 1st-line treatment	35/61	57.4%	5/9	55.6%	0.92
Patients’ characteristics at relapse	VLRs but not EXLRs (5–20 years)		EXLRs (>20 years)		*p*-value
Age ≥ 65 years	11/61	18.0%	4/9	44.4%	0.071
B-symptoms	17/59	28.8%	2/8	25.0%	0.82
Extranodal disease	16/58	27.6%	0/8	0%	0.088
Anemia	21/57	36.8%	1/8	12.5%	0.173
Stage III/IV	27/59	45.8%	2/8	25.0%	0.266
Reinduction	8/61	13.1%	3/8	37.5%	0.071
ASCT intended	14/61	23.0%	0/9	0%	0.108

IPS: international prognostic score; MC: mixed cellularity; ESR: erythrocyte sedimentation rate; RT: radiotherapy; ASCT: autologous stem cell transplantation.

## Data Availability

Data available on request due to privacy.
